# Integrated photothermal decontamination device for N95 respirators

**DOI:** 10.1038/s41598-020-80908-8

**Published:** 2021-01-19

**Authors:** Marcelo Muñoz, Maxime Comtois-Bona, David Cortes, Cagla Eren Cimenci, Qiujiang Du, Collin Thompson, Juan David Figueroa, Vivian Franklin, Peter Liu, Emilio I. Alarcon

**Affiliations:** 1 Division of Cardiac Surgery, University of Ottawa Heart Institute, 40 Ruskin Street, Ottawa, ON K1Y4W7 Canada; 2 Biochemistry, Microbiology, and Immunology, Faculty of Medicine, University of Ottawa, 451 Smyth Road, Ottawa, ON K1H8M5 Canada; 3 Biomedical Mechanical Engineering, University of Ottawa, 800 King Edward Ave, Ottawa, ON K1N6N5 Canada; 4 Cardiac Function Laboratory, University of Ottawa Heart Institute, 40 Ruskin Street, Ottawa, ON K1Y4W7 Canada; 5 Cellular and Molecular Medicine, Faculty of Medicine, University of Ottawa, Ottawa, ON K1H8M5 Canada; 6 Occupational Health, Safety and Biosafety, University of Ottawa Heart Institute, 40 Ruskin street, Ottawa, ON K1Y4W7 Canada; 7 Laboratory Research Resources, Office of Research Services, University of Ottawa Heart Institute, 40 Ruskin Street, Ottawa, ON K1Y4W7 Canada

**Keywords:** Disease prevention, Biomedical engineering

## Abstract

The severe acute respiratory syndrome coronavirus 2 (SARS-CoV-2) responsible for the COVID-19 global pandemic has infected over 25 million people worldwide and resulted in the death of millions. The COVID-19 pandemic has also resulted in a shortage of personal protective equipment (PPE) in many regions around the world, particularly in middle- and low-income countries. The shortages of PPE, such as N95 respirators, is something that will persist until an effective vaccine is made available. Thus, devices that while being easy to operate can also be rapidly deployed in health centers, and long-term residences without the need for major structural overhaul are instrumental to sustainably use N95 respirators. In this report, we present the design and validation of a decontamination device that combines UV-C & B irradiation with mild-temperature treatment. The device can decontaminate up to 20 masks in a cycle of < 30 min. The decontamination process did not damage or reduce the filtering capacity of the masks. Further, the efficacy of the device to eliminate microbes and viruses from the masks was also evaluated. The photothermal treatment of our device was capable of eradicating > 99.9999% of the bacteria and > 99.99% of the virus tested.

## Introduction

Since the end of 2019 and throughout of 2020, the world has been facing a global pandemic caused by the severe acute respiratory syndrome coronavirus 2 (SARS-CoV-2)^1^. Globally, over 25 million confirmed cases have been reported, with over 800,000 deaths^2,3^, numbers which are expected to increase. One of the many concerns and challenges that many countries such as the United States, Canada, and the United Kingdom have faced is the shortage in Personal Protective Equipment (PPE) for frontline healthcare workers^4^. The fluidity of the pandemic progression will most likely extend the PPE shortage to other regions in the globe, as now is the case of Central and South America^5^. This shortage of PPE puts at risk both healthcare professionals and patients^4^. One of the PPE that has been in high demand since the beginning of the outbreak are N95 respirators. These respirators, or masks, help reducing the spread of the virus and protect frontline workers that are treating COVID-19 patients^6^.

Even though N95 respirators are intended for single-use; the exponential consumption of N95 masks has brought the need for finding effective and safe decontamination protocols that can increase the number of uses of the respirators, without damaging the physical integrity and filtering properties^4,6–8^. The current literature reports different methods for the decontamination of face respirators that include the use of heat^9–14^, microwave-generated steam^9,10,12–15^, hydrogen peroxide vapor (HPV)^13,16–18^, ethylene oxide^13,16^, sodium hypochlorite^16^, ethanol^19^, bleach^19^, and UV light^8–10,12–14,16,19–21^. Bacteria and viruses can be deactivated using UV irradiation, with several studies reported using UVC for decontamination^8,21,22^. This deactivation takes place via the production of free radicals that are formed upon the direct interaction of light with the organism’s biomolecules, mainly DNA^23^. However, some species have demonstrated to be resistant to UV and could remain active even after irradiation. Thus, thermal deactivation has been widely use as alternative for eradicating viruses and bacteria^24^. Although the exact mechanism how thermal energy deactivates those organisms are diverse, destabilization of the organism structure via protein denaturation and membrane disruptor are plausible explanations^25^. The primary advantage of using methods such as UV-C and/or heat (in conjunction or alone), when compared to other methods, is that operational personnel can be easily trained, deployed, and specialized facilities are not required for its application. This provides an exceptional advantage for rapid emergency deployment ^13,14^, implementation in non-specialized healthcare centers, or in remote areas. However, for most UV based decontamination protocols, there is a need for using large spaces (entire rooms) and the unintentional UV exposure to the operator is a drawback. The current literature reports on technologies that have used UV-C (0.5–95 J/cm^2^) or temperature (50–75 °C) for eradicating influenza virus from N95 masks, resulting in extended life of the masks^8–10,12,13,16,20,21^. Furthermore, it has been recently reported that SARS-CoV-2 can be inactivated at temperatures ranging from 56 to 70 °C^26^.

**Figure 1:**
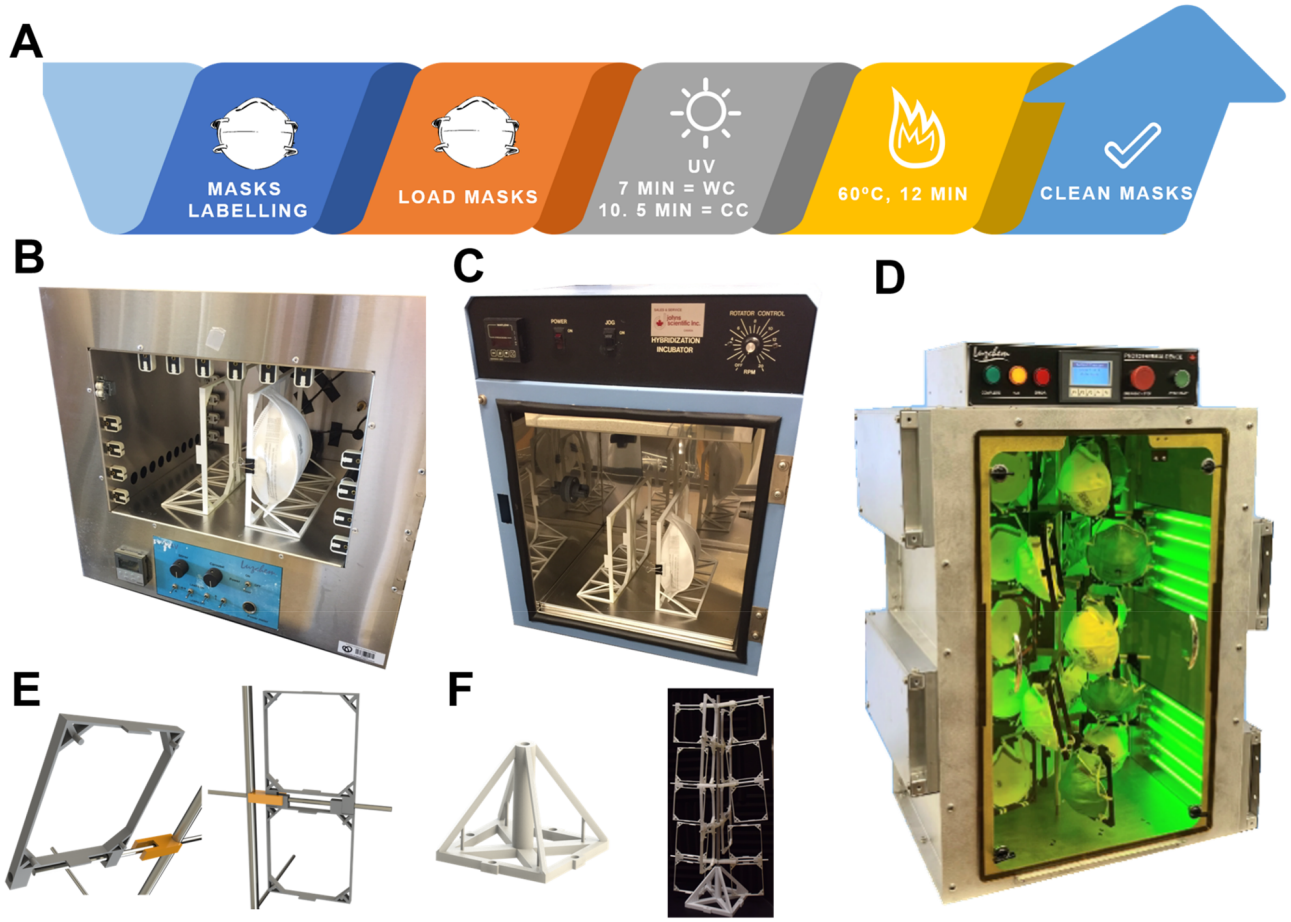
N95 Photothermal decontamination device. (**A**) Decontamination cycle. The masks are labeled, located in the mask holders and center shaft, placed inside of the device, exposed to UV light followed by heat exposure. WC = White Cycle is when UV is used for 7 min, CC = Color Cycle is when UV is used for 10.5 min. (**B**) Photoreactor LZC 4, Luzchem Research Inc., originally used to irradiate up to 4 masks in a semi-manual cycle. (**C**) initial heat application device in prewarmed oven at 60 °C used to treated up to 4 masks per cycle. (**D**) A commercial device designed and manufactured by Luzchem Research Inc, in operation. (**E**) The assembly of the mask holders and location of masks is safely done using a 3D printed shaft support outside of the device to prevent UV exposure and contact with high-temperature surfaces. (**F**) 3D printed mask stand holder, that allows easily loading of the masks into the shaft.

However, the current protocols for using temperature or UV light as decontamination agents are non-standardized and most use UV-C light alone, which does not penetrate the inner layers of the mask^8^, or in the case of static UV-C systems, shadowing within the mask could be detrimental in the procedure (*e.g.* foldable N95 masks 1870 + , see Fig. S1). Attempts for using temperature alone have also failed as the structural integrity of the N95 respirators result severely compromised^27^. Because of this, we sought to develop a device that combines the use of UV-C & B, deeper light penetration, and mild temperatures to effectively decontaminate N95 masks. This all-in-one device allows for automated decontamination using both UV-C & B and heat consecutively. The design of the decontamination device allows the user to safely load up to 20 masks and decontaminate them in an automated device that irradiates the masks with a total irradiation of 1.8 J/cm^2^ followed by heat treatment at 60 °C for 12 min (Luzchem Research Inc, Canada). The device has multiple central shafts such that, while one set of masks is being decontaminated, the operator can load and prepare a second and third set of masks (see Fig. 1).

The performance of the device for decontaminating N95 respirators was assessed using fit testing, filtration efficiency, physical inspection, microbial survival, and virus survival post decontamination experiments. Structural integrity of the masks after photothermal decontamination was also evaluated after three cycles, as well as compared with masks subjected to one cycle of autoclaving. Microbial survival tests were performed with *S. Epidermis, P. Aeruginosa,* and *G. stearothermophilus.* Lentivirus, an enveloped virus, bearing a GFP reporter was used as a surrogate model for SARS-CoV-2. Furthermore, a layout and working protocol were developed in order to decontaminate the masks (see Scheme S1).

## Results and discussion

### Decontamination device

It has been reported that the use of UV-C light and high temperature can effectively eliminate coronaviruses^8–10,12,13,16,20,21^. However, most of these protocols and devices make use of either UV-C or heat alone. Since the fine balance between virus eradication and bacteria killing is critical for safely reusing N95 respirators; we set out for developing a technology that combines both temperature and UV irradiation. The initial prototyping involved the use of separated irradiation and temperature devices, see Fig. 1B,C. This non-automatized decontamination process was able to only load 4 masks per cycle in bench size instruments. Further, since the mask holders remained static, we had to add a step where the irradiation was turned off and the masks positions exchanged to minimize the “shadowed” areas. Apart from the non-homogenous irradiation, we also identified a potential risk of cross contamination when the respirators were transferred between devices. Also, reducing potential risks to the operator, such as UV exposure, as well with a much larger loading capabilities, led to the development of a fully automatized Photothermal Decontamination Device (LPD) by Luzchem Research Inc., see Fig. 1D. The LPD system is able to decontaminate up to 20 masks under 30 min. The decontamination process uses a proprietary combination of UV-C and UV-B irradiation (2.2 J/cm^2^), which maximizes light penetration within the N95 respirators. Further, LPD incorporates irradiation protocols with different time lengths specifically designed for colored N95 respirators. Namely, “White Cycle” (WC, 7 min UV), and “Color Cycle” (CC, 10.5 min UV). Immediately after the irradiation cycles are completed, a temperature treatment (60 °C) for 12 min is used, as shown in Fig. 1A. The short irradiation times used in the LPD system allows for up to 18 months of lifetime of the UV lamps (calculated on 8-h non-stop cycles), which is equivalent to + 300 N95 respirators decontaminated per day. Also, the design of the LPD system allowed for minimal ozone accumulation (≤ 0.02 ppm), which is lower than the hazard level for humans (0.1 ppm, OSHA)^28^.

**Figure 2:**
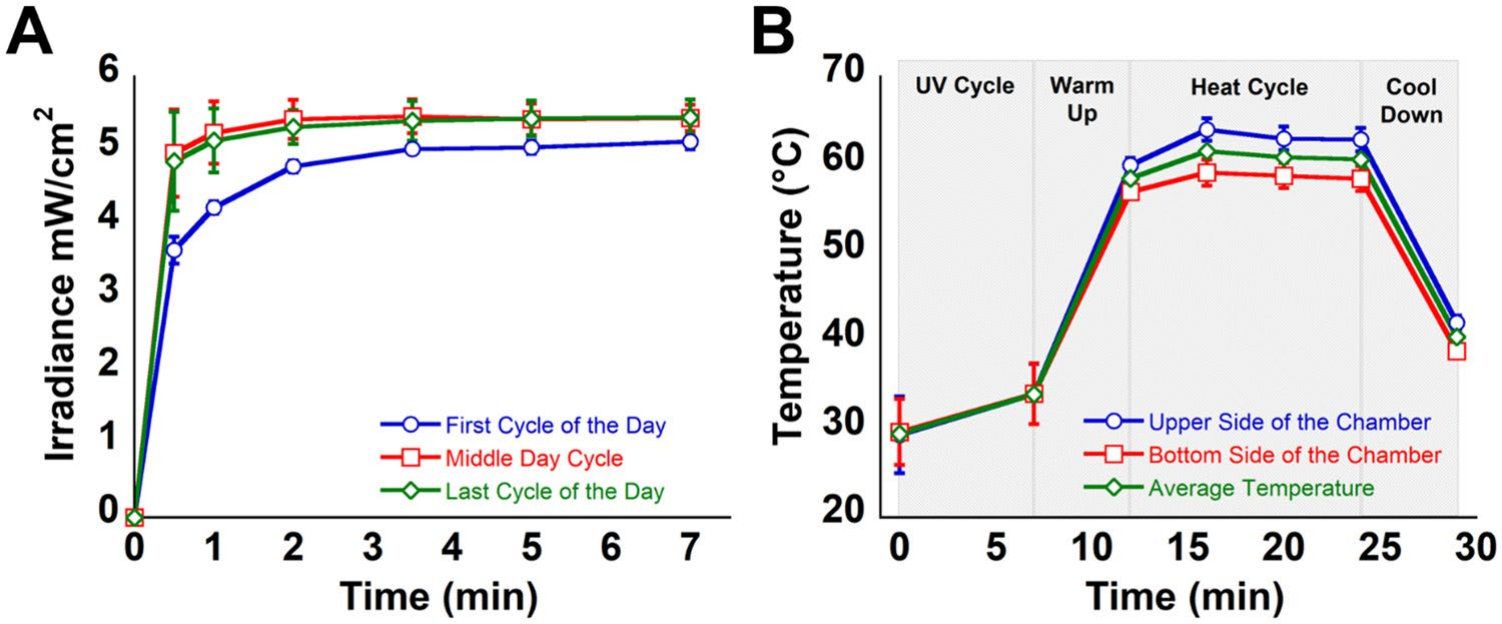
Irradiance and temperature characterization of the device. Irradiance and Temperature validation of the device were measured after 24 h of use. A day cycle is assumed as 8 h of continuous operation. (**A**) For 5 days, irradiance was measured in a center point of the rotating shaft at different times of the day (details in the graph). (**B**) For 5 days, the temperature inside the device was monitored in the upper and lower part of the chamber during the first cycle of the day. Data represent the average point at each time of the different days.

While maintaining a relatively small footprint and light weight, the LPD device contains 4 UV-C/B panels and a heating system that increases the inner temperature, an exhaust for the cooling of the device post-treatment, and a digital panel through which the user can control the device (Fig. 1D). To ensure homogeneous UV exposure of the masks, a rotating center shaft and mask holders were designed such that the masks are homogeneously irradiated (Fig. 1D). An acrylic UV filtering door allows for real time monitoring of the masks during the decontamination process. Videos of the system in operation are available in the Supplementary Information (Videos S1 & S2). The UV dosage and temperature of the device was tested at different points during a regular decontamination day. On Fig. 2A, it shows the irradiance measured inside the device at different timepoints. The first irradiation of the day delivers ≈ 1.8 J/cm^2^ (area under the curve—First cycle of the day) compare to the other total dose, that stabilizes at ≈ 2.2 J/cm^2^ (middle and last cycle of the day). This indicates the need of the device for having a pre-warming cycle. UV-C photosensitive strips were placed on different spots of the mask holders in order to quantify the irradiation homogeneity inside of the device. The results obtained showed that the rotation of the holders inside the LPD system was sufficient to accomplish homogenous irradiation of the masks with dosages between ≈ 2.0 and 2.7 J/cm^2^ (Fig. S2). The temperature profile inside the LPD was measured at different positions (top and bottom of the decontamination chamber, Fig. 2B), and showed that the inner temperature reached 60 °C in approximately 5 min after the UV cycle and remained stable at 60.0 ± 2.0  °C for the remaining of the decontamination cycle.

**Figure 3:**
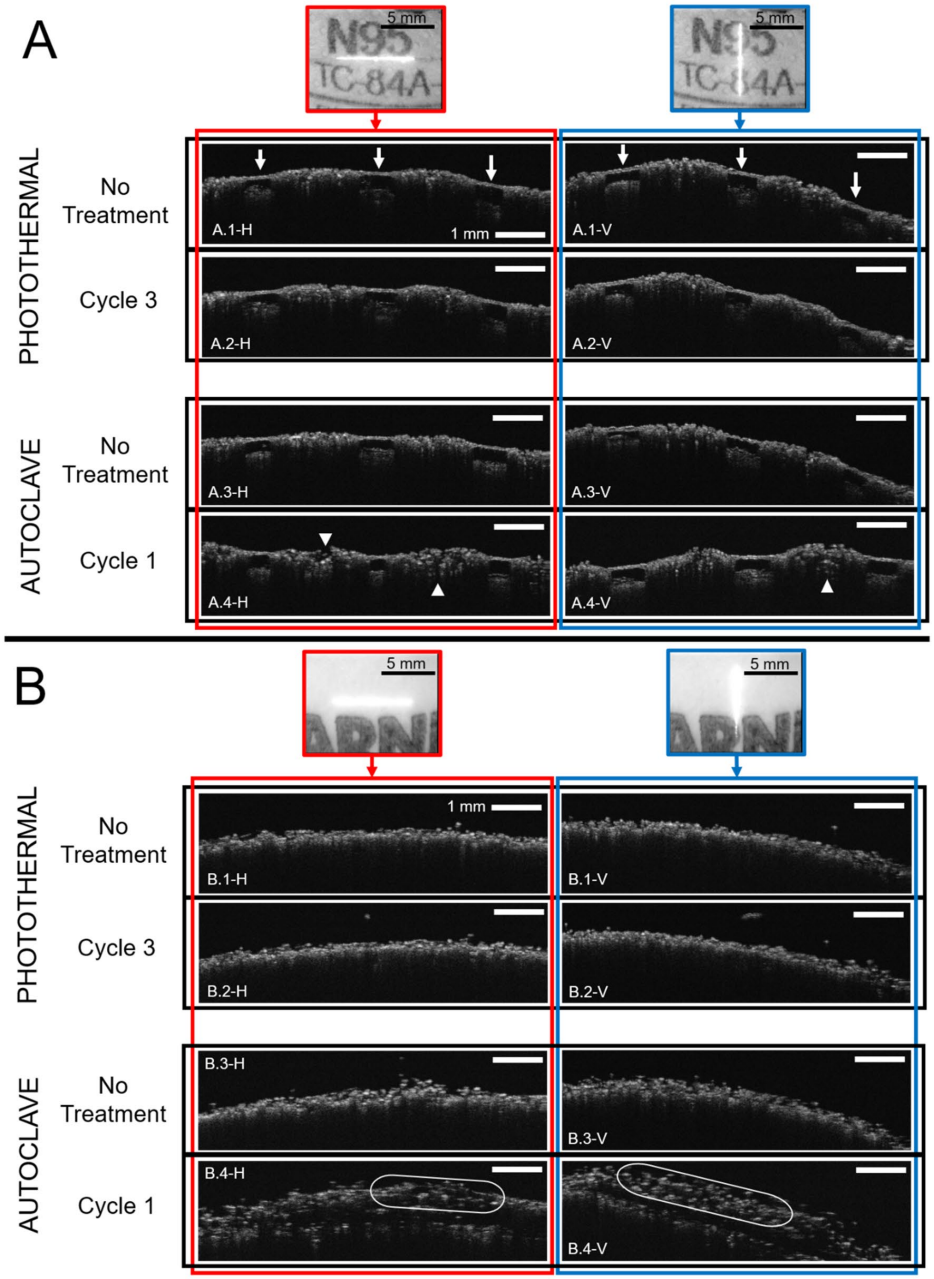
Optical Coherence Tomography (OCT) of N95 masks. (**A**) 1860S N95 and (**B**) 8210 N95 masks evaluated through a horizontal (H) and vertical (V) linear OCT B-scan (7 × 2 line length/depth mm). At the top of every panel, the area of scan is highlighted. Each scan was measured at the same position in each condition. Arrows indicate porous areas of the 1860S N95 mask. Head arrows or ellipses indicate qualitative significant deterioration of the layers of the mask compared to the control.

Once the light dosage and temperature tests were completed, the device was tested using multiple N95 masks at the University of Ottawa Heart Institute (Scheme S1). Since not all N95 masks have the same size and shape, the mask holders were designed such that masks of different sizes and shapes could be decontaminated. The holders were designed to allow homogeneous UV irradiation (Fig. 1E) and 3D printed with acrylonitrile butadiene styrene (ABS) filament.

### Mask holders and center shaft support design

Once 3D printed, the mask holders can be safely attached on each support shaft using an easy-to-assemble, 3D printed assembling system (Fig. 1E and S3). This system allows for the assembly of two vertically aligned mask holders per support shaft, thus effectively utilizing the space within the device to decontaminate more masks per cycle. The center shaft can be easily removed from the device using a push-button system, such that the loading and unloading of the masks can be done outside of the device, reducing the risk of UV light exposure or the contact with high-temperature surfaces by the operator. Multiple center-shafts were designed for each device, such that the decontamination process was made time-efficient, and the operator could load a set of masks, while another set was being decontaminated. To ease the loading and unloading of the masks, a support for the center shaft was designed and 3D printed using polylactic acid (PLA) filament. The center shaft can be safely located on the support and the user can assemble and load the masks as shown in Fig. 1F. A commercial device designed and manufactured by Luzchem Research Inc. in operation is shown in Fig. 1D.

### Validation of decontamination cycles

The impact of the photothermal decontamination on the filtering capacity, fit of the masks, filtration efficiency, physical integrity, microbial, and virus survival were also examined.

#### Physical inspection

In this study, we tested different types of masks available in our Health Care Centre. For the purposes of our study the masks selected were classified in the categories described in Table 1. Picture descriptions of the masks can be found in Supplementary Information, Fig. S1. First, the physical integrity of the masks (visual inspection for physical damage, integrity of the elastic bands, and odor inspection) was evaluated after each decontamination cycle (Table 2). A scoring system from 0 (not useful) to 3 (no changes) was assigned to each parameter by a blind independent individual. From the physical inspection, all the masks showed acceptable physical evaluation for over 3 decontamination cycles.

**Table 1:** Type of 3 M N95 masks and specifications.

Type of mask	Specifications
Foldable and non-foldable	3 M 1870 + is a foldable mask^24^
Porous layers	3 M 1870 + , 3 M 1860^25^ and 3 M 1860S^26^ have diamond shape spaces (like porous) between the first and the second layer
Big and small sizes	3 M 1870 + , 3 M 8210^27^, and 3 M 1860 are considered large masks. The 3 M 1860S is the small version of 3 M 1860
Colour	1860 and 3 M 1860S are fabric coloured on the first layer; the other masks have no colour

**Table 2:** Physical inspection of N95 masks after 3 cycles of decontamination.

Mask model	Cycle 1				Cycle 2				Cycle 3			
	1870 +	8210	1860	1860S	1870 +	8210	1860	1860S	1870 +	8210	1860	1860S
Visual inspection	3	3	3	3	3	3	3	3	3	3	3	3
Elastic band Test	3	3	3	3	3	3	3	3	3	2	3	3
Odor inspection	3	3	3	3	3	3	3	3	3	2 (†)	2 (†)	2 (†)

Qualitative visual inspection was focused on signs of burns and deformation. The integrity of the elastic bands was carried out by stretching the bands from 2 to 6 cm. The odor inspection was done by the tester to identify ozone or burnt smells. The scoring rank was: 3 = No changes with respect to control (before decontamination), 2 = small changes, it does not compromise the physical integrity of the masks, 1 = significant changes (borderline), and 0 = not usable masks. (†) = 1-h post-decontamination score came back to 3.

Visual inspection included inspection of burn areas of the mask. Treatment with UV light and high temperature did not deteriorate the surface of the masks. One of the visual inspections included the metal nose attachment, since it has been reported that this piece might be susceptible to damage during other decontamination methods^14^. None of the masks tested showed any physical deterioration, nose metal damage, or had strong remaining odours. However, after the third cycle some of the models had detectable odour; the odour was not detectable after 1-h post-decontamination (see Table 2). Further decontamination cycles, up to 50, results in a deterioration in the elastic band that was more evident in the 50^th^ cycle alongside with a more persistent odour (see Table S1). However, at 10 and 25 cycles of decontamination, the tested N95 respirators showed comparable properties to those displayed in Table 2.

#### Fit testing and filtration capacity

Assessing the filtration capabilities of the mask after each decontamination cycle is critical to demonstrate the ability of the mask to filter sub-micron particles and to mimic real conditions of mask use. The N95 respirators are characterized for particle filtration > 95%. To evaluate the filtration capabilities of the mask after each cycle, five models of N95 masks were fit tested for up to 3 decontamination cycles (Fig. S4). An overall fitting score above 100 is equivalent to superior filtration capacity of the mask. In all cases, fitting scores were above 100. Furthermore, filtration of 0.075 μm particles were measured (Table 3, raw data included in Table S2) and it was observed that all masks filtered > 99% of the particles after 3 decontamination cycles.

**Table 3:** Filtration Efficiency of N95 mask after 3 cycles.

	1870 +	8210 (CC)	1860
Filtration efficiency	99.3 ± 0.208%	99.6 ± 0.208%	99.9 ± 0.100%

Two types of N95 masks were treated with 3 white cycles (1870 + and 1860) and one type of mask (8210) was treated with 3 cycles of Colour Cycle (CC). The masks were evaluated for filtration efficiency as per *NIOSH N95 42 CFR* (See methodology), n = 3.

As an exploratory study, further decontamination of N95 respirators was carried out for 10, 25 and 50 cycles. The results indicate that our photothermal decontamination does not elicit changes in fit testing up to 50 cycles (Table S3).

#### Structural integrity

To evaluate the structural integrity of the masks after each decontamination cycle, Optical Coherence Tomography (OCT) analyses were carried out. This non-destructive technique allows for a 10 μm resolution or less into the penetration layers^29^. A light source of 820 nm from the OCT allowed for the imaging of the first layers of the N95 masks after each cycle. In addition, for comparison purposes, some masks were cleaned in a wet autoclave cycle and imaged after one autoclave cycle. Two models of N95 masks (1860S and 8210) were subjected to 3 cycles of photothermal decontamination (1860S used Color Cycle, and 8210 used White Cycle) or to one cycle of autoclaving. Detailed views of the scanning site for each mask (7 mm length) and the scans obtained of each mask in a horizontal (H) and vertical (V) linear scan are shown in Fig. 3. Measurements of the layer thickness at the porous area (1860S), first layer density at porous areas (1860S), and first layer density in non-porous areas (1860S and 8210) were calculated on ImageJ (see methodology) and shown in Table 4.

**Table 4:** Analysis of N95 first layer thickness and density by OCT.

Mask model	3 M 1860S			3 M 8210
	Layer thickness at porous area (μm)	Density of first layer at porous area (%)	Density of first layer at non-porous area (%)	Density of first layer (%)
**Photothermal decontamination**				
No treatment		83.07 ± 10.68	97.82 ± 2.52	87.74 ± 6.49
1st Cycle	92.29 ± 11.50	98.37 ± 1.82	90.34 ± 9.33	97.34 ± 1.99
2nd Cycle	85.31 ± 12.62	99.02 ± 0.15	85.30 ± 3.41	95.12 ± 1.98
3rd Cycle	86.39 ± 9,78	99.04 ± 1.21	86.26 ± 5.44	96.40 ± 1.10
**Autoclave**				
No treatment	78.57 ± 13.97	98.41 ± 0.85	90.48 ± 1.34	87.82 ± 6.46
1st Cycle	87.49 ± 12.98	99.46 ± 0.45	66.83 ± 4.24*	47.26 ± 16.96**

Semi-quantitative measurements of the first layer of the 3 M 1860S N95 and 3 M 8210 N95 masks. 3 M 1860S mask integrity was assessed at the porous area (thickness and density) and at the non-porous area (density). 3 M 8210 mask integrity was assessed as the density at the first layer. For further details see methodology. * = significant difference (*p* < 0.05), and ** = significant difference (*p* < 0.01), compared to the respectively “No Treatment” results, calculated from t-test analysis (n = 3).

**Figure 4:**
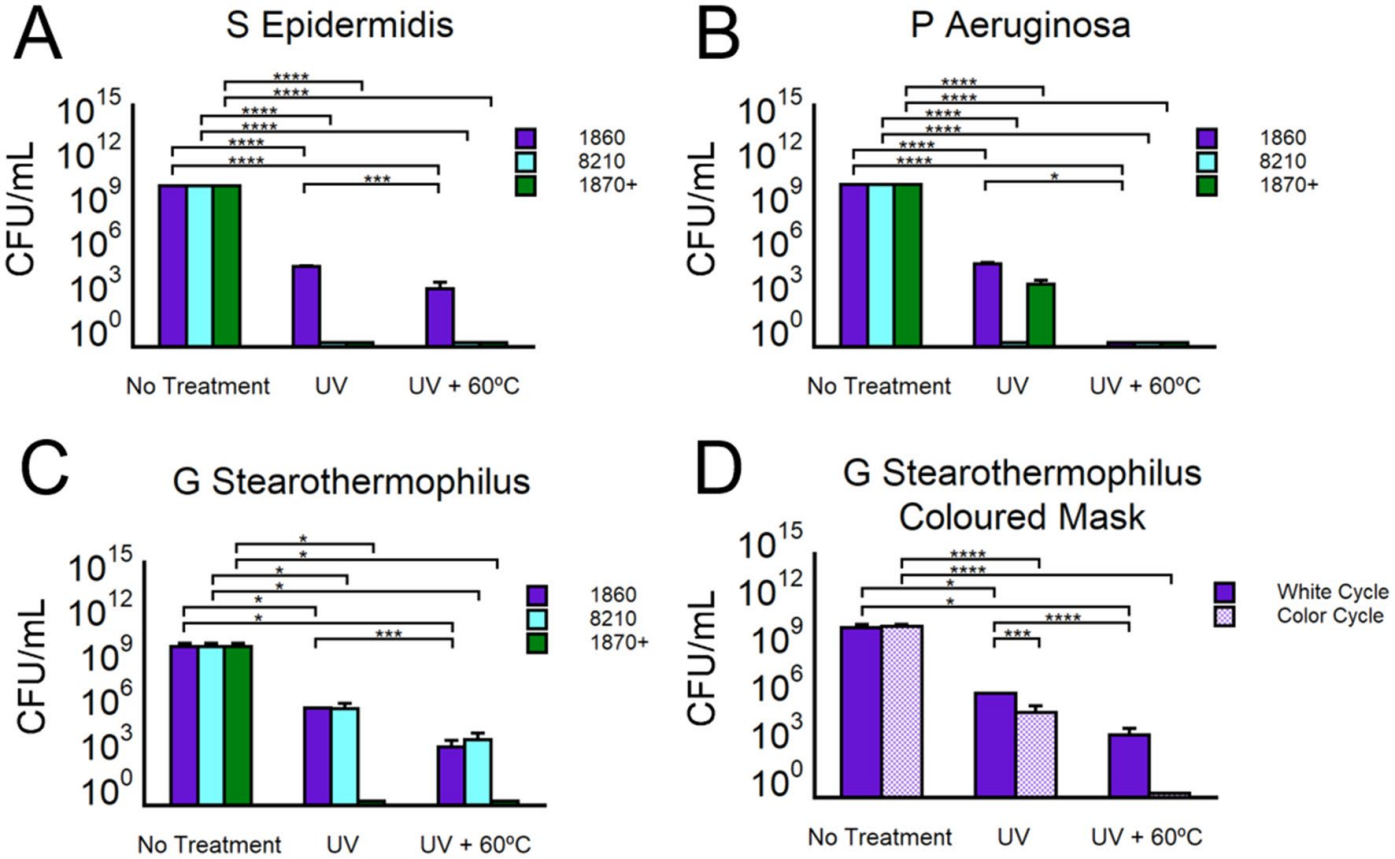
Microbiological Validation of the decontamination process. Photothermal decontamination process under White Cycle (7 min UV + 12 min 60 °C) with N95 masks: 1860, 8210, and 1870 + on (**A**) *S. Epidermidis*, (**B**) *P. Aeruginosa*, and (**C**) *G. Stearothermophilus.* (**D**) Comparison of white cycle and color cycle decontamination (10.5 min UV + 12 min 60 °C) on the coloured N95 model (1860) inoculated with *G. Stearothermophilus*. For all the experiments, the different experimental groups correspond to: No Treatment (bacteria infected on the masks and transferred to agar plates), UV (same as No Treatment, but adding the UV step of 7 min in the white cycle, or 10.5 min in the coloured cycle), and UV + 60 °C (same as UV, but adding an step of 60 °C for 12 min after UV). All the groups had a sample size of n = 3. (*) indicates p values < 0.05, (**) indicates p values < 0.01, (***) indicates p values < 0.001, and (****) indicates p values < 0.0001 calculated from t-test analysis (n = 3).

Autoclaving of N95 respirators has been shown to produce nose metal detachment and loss in filtration properties^27^, as confirmed by the data showed in Fig. 3. The 1860S masks have porous areas formed between the first and the second layer (Fig. 3). These pores can be easily seen in the OCT scan of Fig. 3-A1.H and A1.V (arrows). After 3 cycles of photothermal decontamination, no significant changes to the physical integrity of the first layers was observed in the OCT images (Fig. 3-A2.H and A2.V). In comparison, after only 1 autoclave cycle, changes within the layers of the masks were observed (arrows heads in Fig. 3-A4.H and A4.V). A semi-quantitative analysis (see methodology) was performed in the porous areas (thickness and density) and in non-porous areas (density). As can be seen in Table 4, the only significant differences before and after decontamination were measured for the 1^st^ cycle of autoclave. 8210 masks are designed without pores between the first and second layer, as can be seen in the OCT scans of Fig. 4-B1.H and B1.V. After 3 cycles of photothermal decontamination, no changes were observed (Fig. 3-B2.H and B2.V, and Table 4). But, after one autoclave wet cycle, the first layer was completely disrupted, resulting in gaps between the layers (Fig. 3-B4.H and B4.V and Table 4). Further decontamination cycles, up to 50, for the 1860S and 8110S respirators does not produce a statistically significant decrease in the structural integrity of the respirators (Fig. S5 and Table S4).

### Microbiological decontamination validation

#### Bacterial decontamination

To assess the ability of the device to decontaminate N95 masks, 3 bacterial strains were used: *Staphylococcus Epidermidis* (*S. Epidermidis*), *Pseudomona Aeruginosa* (*P. Aeruginosa*), and *Geobacillus Stearothermophilus* (*G. Stearothermophilus*). The first two species represent the two type of gram-positive and gram-negative bacteria, respectively. Additionally, both are found on serious or chronic infectious disease clinical settings^30,31^ and both species can generate multi-resistant properties against antibiotics^32,33^. *G. Stearothermophilus* is a standard bacterial strain used for validation of wet sterilization cycles^27,34^. Its capabilities as a bacteria thermophila (it is able to growth at 55 °C)^35^ make it an ideal strain to determine the efficacy of our device to effectively decontaminate PPE. Furthermore, a qualitative biological test was evaluated with spore strips of the strain *Bacillus Pumilus*, which is a reference standard microorganism test for high radiation devices^36–38^ (see Fig. S6).

We selected three mask models: 1860, 8210, and 1870 + . Each mask was inoculated with a bacterial concentration of ≈10^10^ CFU/mL. This concentration value exceeds the standard used to certify face masks in the industry (5 × 10^5^ CFU/mL, ASTM F2101)^39^. The bacteria were spread homogenously on the surface and let absorbed into the inner layers of the mask for 1-h prior to testing. The inoculated samples with and without the decontamination cycles were transferred to plates containing sterile bacteria culture media and incubated for 1 h. Then, 10 µl of that solution was plated for counting analysis (Fig. 4A–C, tabulated in Table S5). For *S. Epidermidis* and *P. Aeruginosa*, the reduction on the masks 8210 and 1870 + was over 8 log_10_ after the full white cycle decontamination. For 1860, the reduction was 6 log_10_ after the UV treatment (White Cycle) then increased to + 8 log_10_ upon the thermal cycle, indicative of the complementary process between UV and temperature (significant difference). For *G. Stearothermophilus*, the results observed were 8 > log_10_ and for the 1870 + masks and 6 > log_10_ for the masks 1860 and 8210. Since the 1860 mask models are pigmented (see Fig. S1), the extended UV irradiation time (CC) showed improvement in bacterial reduction (Fig. 4D). A significant difference was observed in the number of colonies that survived between the CC and WC groups, Fig. 4D.

Additional experiments for *G. Stearothermophilus* with only temperature treatment (incubated at 60 °C for 12 min) showed almost no effect on the bacterial growth, see Fig. S7. This is compatible with the survival of these species at such high temperatures (optimal incubation temperature 55 °C). This result illustrates on the relevance of having the combinatory cycle of UV and temperature as an effective and rapid route for safe decontamination of N95 respirators.

#### Viral decontamination

**Figure 5:**
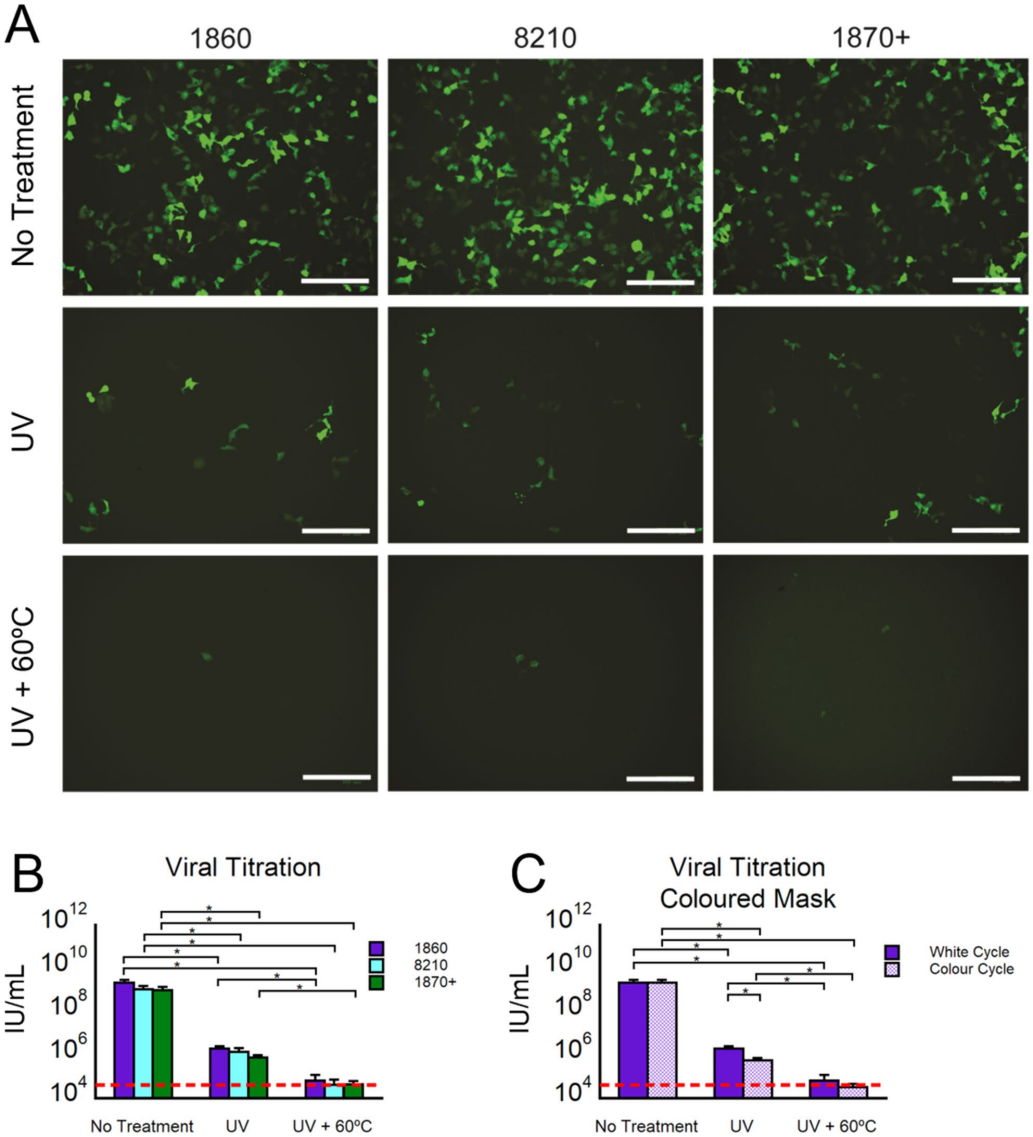
Viral Validation of the decontamination process. Photothermal decontamination process under White Cycle (7 min UV + 12 min 60 °C) or Colour Cycle (10.5 min UV + 12 min 60 °C) with N95 masks 1860, 8210, and 1870 + . (**A**) Representative fluorescent images of GFP + virus-infected 293A cells experiment on each group. (**B**) Virus titer of infected 293A cells. Analysis was performed after 72 h of incubation for the different N95 mask models with White Cycle. Red line indicates limit of detection of the methodology. (**C**) For the 1860 a comparison study of White and Color Cycle decontamination was performed. Red line indicates limit of detection of the methodology. For all the experiments, the different experimental groups correspond to: No Treatment (virus infected on the masks and transferred to 293A cells), UV (same as No Treatment, but adding the UV step of 7 min in the white cycle, or 10.5 min in the coloured cycle), and UV + 60 °C (same as UV, but adding an step of 60 °C for 12 min after UV). All the groups are samples n = 3. (*) indicate p values < 0.05, (**) indicate p values < 0.01, (***) indicate p values < 0.001, and (****) indicate p values < 0.0001 calculated from t-test analysis (n = 3).

The effects of the decontamination process on viral infection were then investigated by testing the infectious ability of the Lentivirus on HEK293A cells upon UV and UV + heat treatments (Fig. 5A–C). As a surrogate virus model for SARS-CoV-2, we used a Lentivirus bearing a green fluorescent protein (GFP) reporter. It has been known that the spikes of different coronaviruses can be pseudotyped in order to create safer experimental models^40,41^. Previous studies showed that HIV-based Lentiviral particles were used as a model in numerous SARS-CoV-2 studies^42,43^. The surface glycoproteins of coronaviruses play an important role in receptor binding and cell entry. This makes Lentivirus a good candidate for our study as they share similar envelope and lipid bilayer surface features.

Our results showed that, similar to the bacteria tests, decontamination cycles resulted in a significant reduction in viral infection. We set up our in vitro infection test plates with a dose of ≈10^9^ IU/mL virus per well (Fig. 5A–C) and we treated such plates either with UV-only or UV + heat treatments. Our results indicate that viral infection was significantly decreased from ≈10^9^ IU/mL to ≈10^4^ IU/mL and < 10^4^ IU/mL in the UV-only group and in the UV + 60 °C treated groups, respectively (Fig. 5B,C, red line is the detection threshold of qPCR, tabulated in Table S6). Overall, Lentivirus without any treatment infected ≈40% of the groups whereas UV-C + 60 °C treatment reduced this effect to < 0.5%. We found no significant differences between mask models vs HEK293A cells infected with Lentivirus. These results indicate that both treatments were equally effective in eliminating the virus when compared to non-treated masks (no significance difference between CC and WC with UV + heat).

## Conclusions

The LPD device combines UV-B and C and a short mild temperature to effectively decontaminate up to 20 masks at a time in less than 30 min. The decontaminated N95 respirators showed no physical damage after 3 cycles, with no significant signs of degradation of the filtering materials or elastic bands. From the fit testing, it was determined that the mask models could undergo up to 3 cycles of decontamination. Additionally, the layer thickness and material density of the masks after decontamination with our device was not impacted after multiple cycles. Exploratory tests increasing the number of decontaminations cycle up to 50 cycles showed that respirators are still suitable for use, although there was a wearing of the elastic bands. Furthermore, our device was able to eliminate 8 log_10_ of *P. Aeruginosa*, *S. epidermidis*, and *G. Stearothermophilus* and 4 log_10_ of our surrogate Lentivirus from within the different mask layers. Overall, the photothermal decontamination device presented here is an attractive and cost-effective tool with a relatively small footprint that allows for a solvent/gas free, and dry decontamination of N95 masks without compromising their macro and microscopic physical integrity and filtering capacity. Further development of N95 respirators could incorporate using activatable materials that absorb in the visible spectrum for allowing for “real-time” sunlight bacterial and viral eradication^44,45^.

## Methods

### CAD modeling and 3D printing

All 3D printed components were designed using computer-aided design (CAD) software (SolidWorks, Dassault Systemes), sliced in Ultimaker Cura 4.6 software, and 3D printed in an Ultimaker S5 (Ultimaker, Netherlands). The 3D mask holders were designed in three main sections: a top bracket, a bottom bracket and a vertical alignment piece to assemble the former two pieces together on the support shaft. The bracket was designed as an open rectangular frame with an available space of 120 mm × 130 mm to accommodates N95 masks of different sizes and shapes. To locate the elastic bands of the masks and prevent shadow generation or entanglement of components, four cylindrical pins were placed at the corners, where the elastic bands are placed during decontamination.

In order to align the two brackets that were placed vertically on the second and third level support shafts, an alignment piece was designed, and 3D printed. This piece was located against the center shaft to prevent rotation about the support shafts, to maintain both upper and lower brackets at a 180˚ angle from each other. The mask holders and alignment piece were 3D printed in ABS (Filaments Canada, Canada) filament with an infill density of 15%, layer height of 0.2 mm, wall thickness of 0.8 mm, printing temperature of 250 ˚C and print bed temperature of 95 ˚C. A brim with a width of 12.0 mm was placed inside and outside the model along with a layer of glue on the glass print bed to prevent warping during printing. The center shaft support was 3D printed in polylactic acid (PLA) filament at an infill density of 20%, layer height of 0.2 mm, 1.2 mm of wall thickness, printing temperature of 215 ˚C, print bed temperature of 60˚C, and support overhang angle of 45˚.

### Photothermal decontamination device (LPD)

The LPD device was developed by Luzchem Research Inc. The device needs of minimal ventilation. The device was certified by CSA (110 V, 60HZ and 10A) with an average energy consumption of 1100 W, which in a 12 h shift will total 13.2 kWh/day.

#### Photothermal decontamination system specifications and stability testing

The device was characterized for UV dose and temperature. The UV dosage of the device was tested during a regular day of use (20 cycles). The irradiance profiles were measured in cycle 1, 10, and 20, see Fig. 2A. Measurements were carried out using a Luzchem Spectroradiometer calibrated for UV irradiation (Smart Sensor L-0487). The head of the sensor was placed at the center of the device and irradiation dosage measured every minute until a plateau in the decontamination was reached, after this, data was acquired every 2 min. Area under the curve is equal to the total energy dose delivered by lamps. Temperature stability and profiles were measured in the top and bottom positions inside the decontamination chamber at the same cycles previously mentioned in this paragraph (Fig. 2B). A multichannel Datalogging Thermomether (Sper Scientific RAM-000887) with 2 K-type thermocouples were used for those measurements. Temperatures were registered at points: UV start, UV finish, Heating start, Temperature Plateau (every 5 min), Heating stop, and finish cool down period. Ozone levels were measured with EZ-1X ECOZONE Ozone Monitor, Serial Number 816946, at 3 different levels inside and outside of the device, during 2 continuous cycles of UV white cycle in each position (14 min of irradiation). In none of the positions evaluated the ozone detection was above 0.02 ppm (limit of detection of the device, see Figs. S8 and S9).

#### Fit testing

The N95 respirator fit testing was completed using the Quantitative Respirator Fit Testing (QNFT) Method with use of the PORTACOUNT Pro + 8038 Respirator Fit Tester, in adherence to the *CSA Standard Z94.4–18—Selection, Use, and Care or Respirators*^46^. Fit testing was carried out following the Occupational Health, Safety, and Biosafety approved protocol of the University of Ottawa Heart Institute approved by the Institutional Review Board. This protocol follows the guidelines for fit testing of N95 respirators in healthcare workers in Canada (*CSA Standard Z94.4–18*). The volunteers signed informed consent were used in the fit testing evaluation. The data were analyzed by an independent observer. Briefly, in an enclosed room, 0.3 µm particles of NaCl are vaporized into the ambient at room temperature. The respirator to be evaluated was punched with a push probe and nut, then and attached to the hose that is connected to the PORTACOUNT Pro + . Under instructions of the evaluator, the person dons the respirator in accordance to the manufactures donning instructions, which also includes performing a User Seal Check. After donning the respirator, the fit testing software in initiated and performs the following exercises: 1. Normal Breathing; 2. Deep Breathing; 3. Turning Head Side to Side; 4. Nod Head Up and Down; 5. Talk Out Loud. In each exercise, the PORTACOUNT Pro + measures the difference in particle concentration of the challenge aerosol (C_out_) and the particle concentrations inside the respirator (C_in_), quantifying that the respirator is performing at the required level of efficiency. Exercise results could range in fit factor values from 1 (all particles run through the respirator) and up to + 200 (most of the particles are filtered by the respirator). If the Overall Fit Factor is > 100, the test is considered as an Overall Pass.

Fit factor (FF) is calculated with the formula:

1 $$FF=\frac{{C}_{B}+{C}_{A}}{2{C}_{R}}$$

where FF = fit factor, CB = particle concentration in the ambient sample before the respirator sample, CA = particle concentration in the ambient sample after the respirator sample, CR = particle concentration in the respirator sample.

### Filtration efficiency

The filtration efficiency was measured under conditions of the standard *NIOSH N95 42 CFR Part 84*. Briefly, masks were preconditioned at 85% humidity for 25 h. After this, masks were transferred to the automated filter tester 8130A (TSI Incorporated, NRC facilities, Ottawa, ON, Canada), under flow of 85 L per minute and loading of 200 mg of NaCl particles of size 0.075 ± 0.02 μm. Original data shown in Table S2.

### Structural integrity

The layer thickness and material density of the masks were calculated from Optical Coherence Tomography (OCT) images analyzed in ImageJ (National Institutes of Health). The analysis of the OCT images was performed in a time domain OCT (Carl Zeiss Meditec, Inc.). Each A scan shown in Fig. 3 consisted of 1024 scans (2 mm deep into the masks) at a speed of 400 Hz, and B scan consisted of 512 scans (7 mm linear length). A line scan was performed in horizontal and vertical orientations. The masks where placed in front of the OCT lens in a perpendicular position through a custom design holder. At the same time of scan acquisition, a photo was acquired to reproduce the same position every time after each cycle. The data was extracted in ascii format and processed in ImageJ. The X:Z ratio was corrected and interpolated (cubic interpolation) to real size. For quantification purposes, each image was processed to remove the noise and thresholding the image to obtain the masks layers in a binary format (Fig. S10A–C). The layer thickness at porous areas (mask 1860S) was measured obtaining vertical linear distance between the upper and lower edge of the layer, every 0.5 μm distance in the x -axis. Every slice was quantified in its length (top to bottom) from the start to the end of pixel intensity (Fig. S10C.1, intensity threshold: 127 of 255 total intensity). All the measurements were tabulated and averaged for statistical analysis. The density of the material at non-porous areas was calculated by selecting an 800 × 100 μm rectangle in a non-porous area of the mask with a distance from the top layer of 50 μm. The percentage area of pixel intensity was calculated versus the total area of the frame (Fig. S10C.2). The density of the material of a porous area was calculated similarly, by drawing a rectangle of 800 × 40 μm in the middle of the layer on the porous area (Fig. S10C.3). The image processing flow is shown in Fig. S10.

### Autoclave

The autoclave cycles were performed in a Ritter M11 UltraClave. Briefly, masks were placed in seal pouches, and exposed to a wet cycle at 132 °C/186 kPa for 15 min. After cooling down and dry in the autoclave, the masks were dried overnight.

### Microbial survival

All experiments were carried out in a BSL2 certified laboratory. Bacteria cultures were obtained from single colonies that were resuspended in 2 mL of 100% LB agar broth and incubated in an orbital shaker incubator for 16–18 h at 200 rpm and incubated at 37 °C and 55 °C for *P. aeruginosa* (PA14) & *S. epidermidis* (ATCC 35,984), and *Geobacillus stearothermophilus* (Wards Science), respectively. Then, solutions of bacteria with concentrations of ≈10^10^ CFU/mL were prepared, 15 µl inoculated onto 5.0 × 1.5 cm pieces of the different tested masks and underwent the photothermal decontamination (in triplicates). The 5.0 × 1.5 cm pieces were incubated in 12 well plates with 2 ml of 10% LB media for 1 h and plated on LB agar and quantified by CFU counting (10 µL per sample). Agar plates were incubated for 12 h and incubated at 37 °C for *P. aeruginosa* & *S. epidermidis,* and 55 °C for *Geobacillus stearothermophilus*, data shown in Table S5.

### Virus survival

All experiments were carried out in a BSL2 certified laboratory. As a surrogate virus variant for SARS-CoV-2, a Lentivirus bearing a GFP reporter gene was used. The lentivirus species we used had encoded a green fluorescent protein reporter (pLL-CMV-rFLuc-T2A-GFP-Puro LENTI-LABELER LENTIVECTOR, LL310VA-1, SBI System Biology) at a viral dosage of 10^9^ IU in 1% BSA in PBS. The calibration curve for the IU vs. Ct value was obtained from the qPCR, using the using the Lentiviral DNA vector included in qPCR Lentivirus Titration Kit (Applied Biological Materials Inc, Canada). The efficiency of the virus was tested on Lentivirus-infected HEK293A cells.

Pieces of the masks (5.0 × 1.5 cm) were inoculated with 15 µl of 10^9^ IU/mL viral solution and were exposed to the photothermal decontamination cycle. After decontamination, the pieces were transferred to 15 ml centrifuge tubes containing 2.5 ml of culture medium (DMEM 10%FBS) and were spun 30 times to elute virus from the mask piece. The media with the extracted virus was used to feed 293A cells at a density of 1 × 10^6^ cells/ml. Cells were cultured for 72 h at 37C, 5% CO2. Green fluorescent protein expression (GFP +) was quantified using fluorescence microscopy with a 488 nm excitation and 525 ± 30 nm detection. The number of IU was determined by extrapolation of the % of GFP + in a calibration curve for the genomic copy number versus Ct value was obtained from the qPCR device, prepared for different concentrations of IU (10^4^–10^9^ IU), raw data shown in Table S6. It is important to note that, the qPCR kit’s lowest measurable limit of detection was determined as 10^4^ IU/ml.

### Statistical analysis

Statistical analyses were carried out in Microsoft Excel version 16.44 with the t-test function for samples with unequal variance.

## Supplementary Information

Supplementary Information.

## Data Availability

All data generated or analyzed in this study are included in the manuscript and the Supplementary materials. Raw data for Figs. 2A,B, 4 and 5 alongside with the STL files for the 3D printed mask holders can be found https://figshare.com/s/a4cfa7c7df4c5f13033f. Additional experimental data that support the findings of this study are available from the authors.
