# Supplementary material for: Integrated photothermal decontamination device for N95 respirators
Source: Sci Rep. 2021 Jan 19;11:1822. doi: 10.1038/s41598-020-80908-8 (PMC7815715; doi:10.1038/s41598-020-80908-8)
Supplement: Supplementary file 1 — Supplementary Information. [file 41598_2020_80908_MOESM1_ESM.pdf]

Electronic Supplementary Information (ESI) for:

Integrated Photothermal Decontamination Device for N95 Respirators

Marcelo Muñoz[1,2], Maxime Comtois-Bona[1,3], David Cortes[1,3], Cagla Eren Cimenci[1,5], Qiujiang Du[4], Collin Thompson[6], Juan David Figueroa[1], Vivian Franklin[7], Peter Liu[4], Emilio I. Alarcon[2,3*]

[1] Division of Cardiac Surgery, University of Ottawa Heart Institute, 40 Ruskin street, Ottawa, ON, K1Y4W7, Canada.

[2] Biochemistry, Microbiology and Immunology, University of Ottawa, 451 Smyth Road, Ottawa, ON, K1H8M5, Canada,

[3] Biomedical Mechanical Engineering, University of Ottawa, 800 King Edward Ave, Ottawa, ON, K1N6N5, Canada.

[4] Cardiac Function Laboratory, University of Ottawa Heart Institute, 40 Ruskin street, Ottawa, ON, K1Y4W7, Canada.

[5] Cellular and Molecular Medicine, Faculty of Medicine, University of Ottawa, Ottawa, ON, K1H8M5, Canada.

[6] Senior Safety Advisor, Occupational Health, Safety and Biosafety, University of Ottawa Heart Institute, 40 Ruskin street, Ottawa, ON, K1Y4W7, Canada.

[7] Laboratory Research Resources, Office of Research Services, University of Ottawa Heart Institute, 40 Ruskin street, Ottawa, ON, K1Y4W7, Canada.

*email: ealarcon@ottawaheart.ca

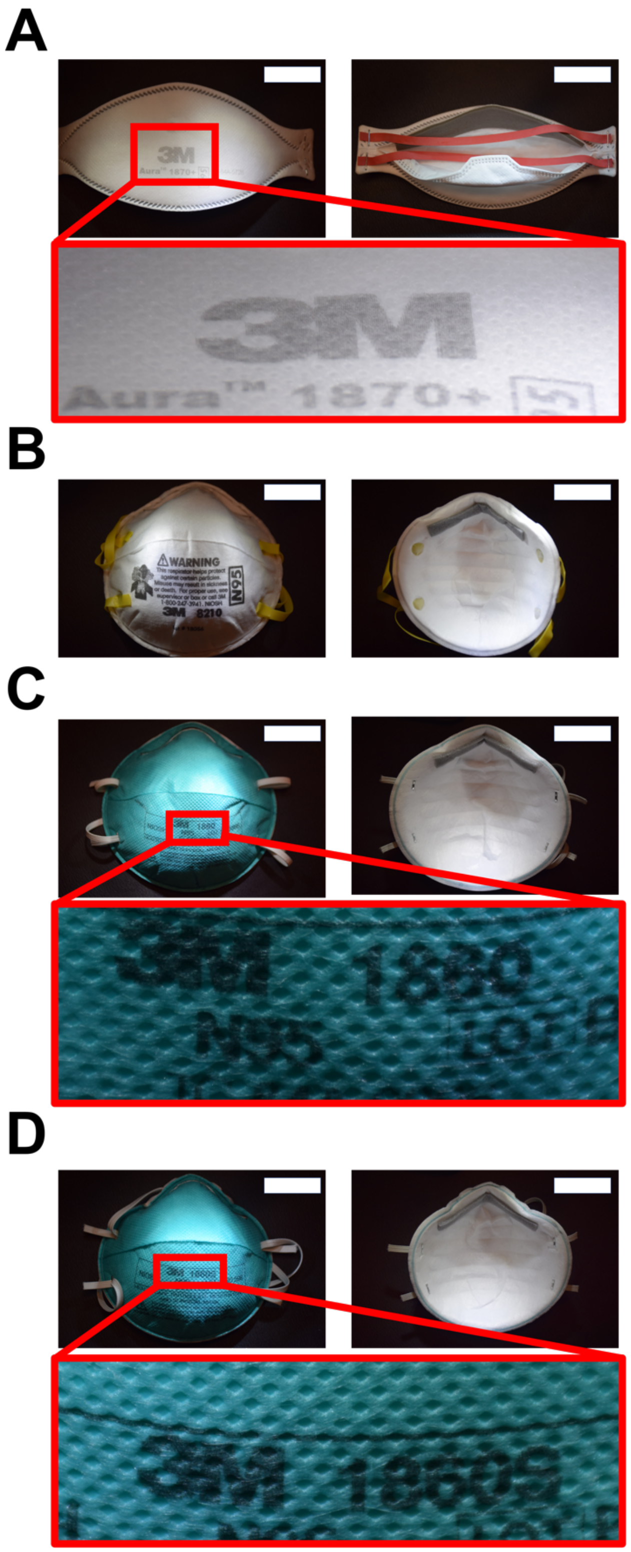

Figure S1. Front and posterior image of N95 masks used in this study. (A) foldable 1870 (B) 8210+, (C) 1860, and (D) 1860S. To highlighted pores at the first layer, zoom is shown. Scale bar 4 cm

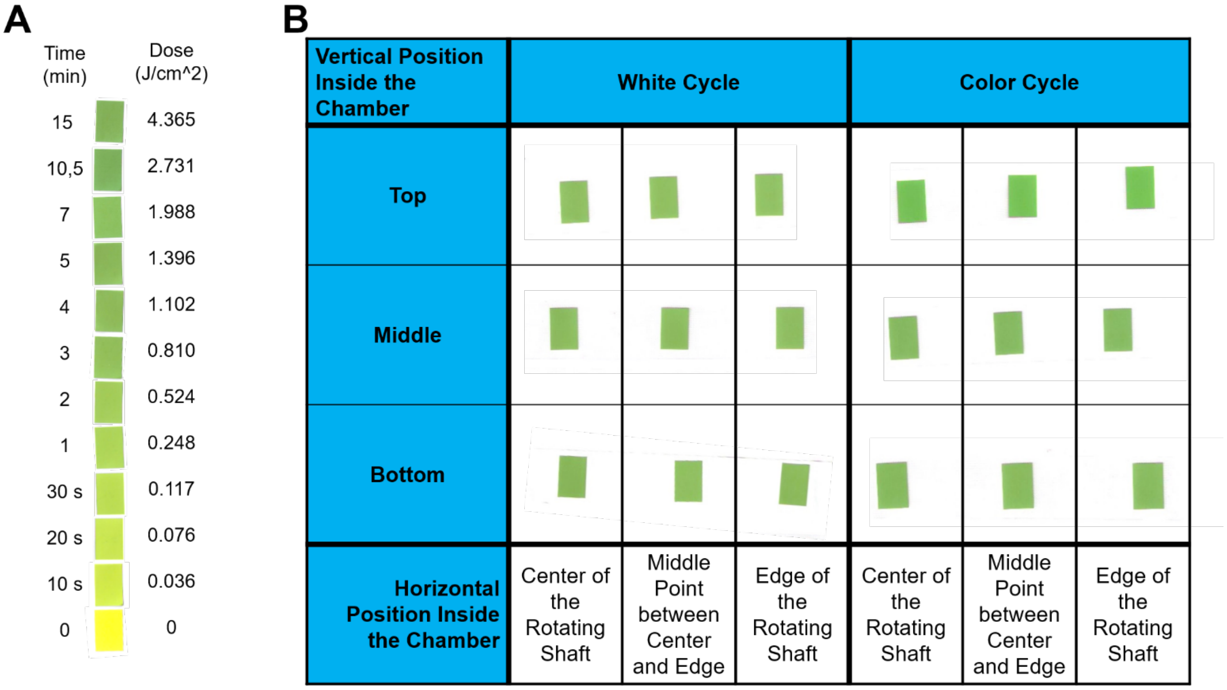

Figure S2. UVC strips qualitative quality control test. UV-C strips (UV-C intensity Labels, catalogue number: N010-004, UV Process Supply, INC.) were used for the test. **(A)** A color change calibration experiment was performed in the device at different time points and irradiance correlation. **(B)** 3 strips were placed in a custom-made device at the same level of the masks at the top, middle, and bottom position inside the chamber. The 3 strips were positioned equidistantly between the center and the edge of the rotating shaft. White Cycle is equivalent only to 7 min UV, and Color Cycle is equivalent only to 10.5 min UV (no heat).

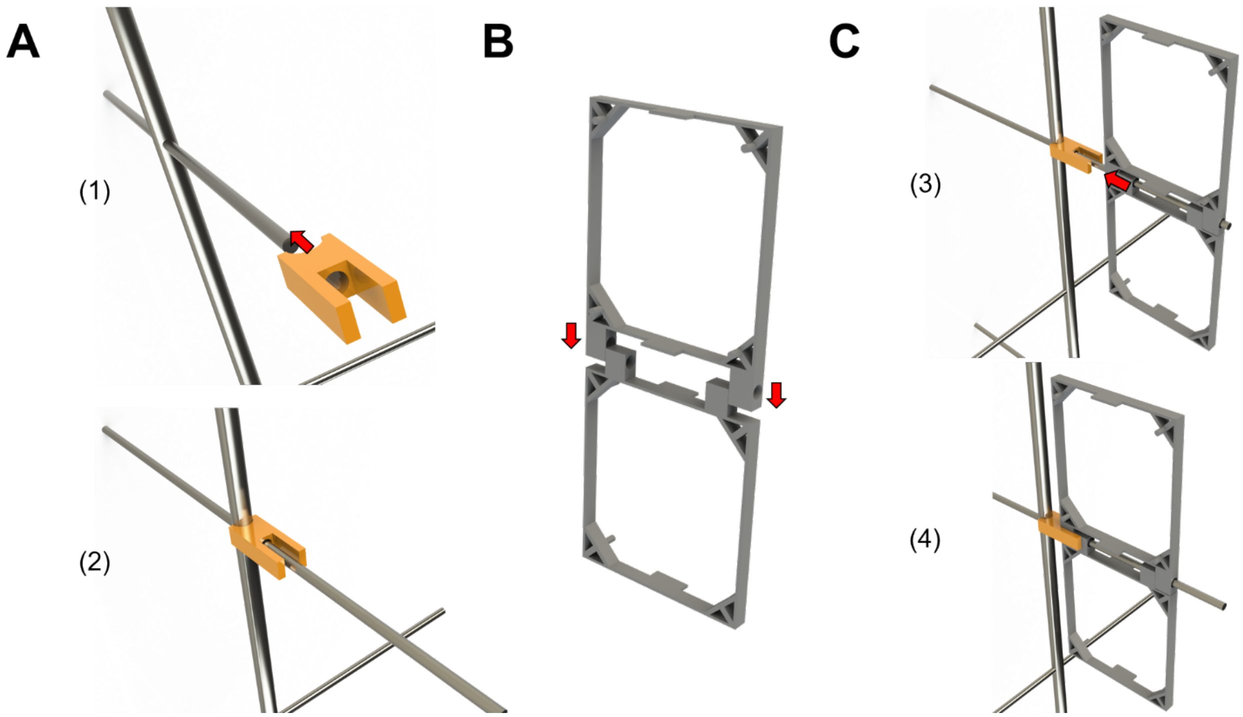

Figure S3. Assembly of the N95 Mask Holder. To assemble the mask holders in the center shaft, first A) the vertical alignment piece is inserted in the support shaft (1) until the semicircular face mates with the center shaft (2). Then, once having the masks mounted on each mask holder (not shown), B) the top and bottom holders are aligned together. C) Maintaining the holders aligned, they are inserted in the support shaft (3) until they meet the plain face of the vertical alignment piece (4).

- N95 Mask Mounting Steps:
  1. For Rigid Mask (1860s, 8210)
     i. Place the mask in the middle of the holder.
     ii. Using the flat hook at the top and bottom, wrap the elastic band around them to secure the mask. Adjust the position of the mask by wrapping the remaining elastic bands around the cylindrical struts at the corners of the support.
     iii. Secure the mask by placing a metal clip between the flat hook and the elastic band.
  2. For Foldable Mask (1870)
     i. Place the side of the masks on the exterior face of the side hook and secure it with a metallic clip.
     ii. Repeat the same process for bottom side of the mask, in order to maintain it open at all times during decontamination.

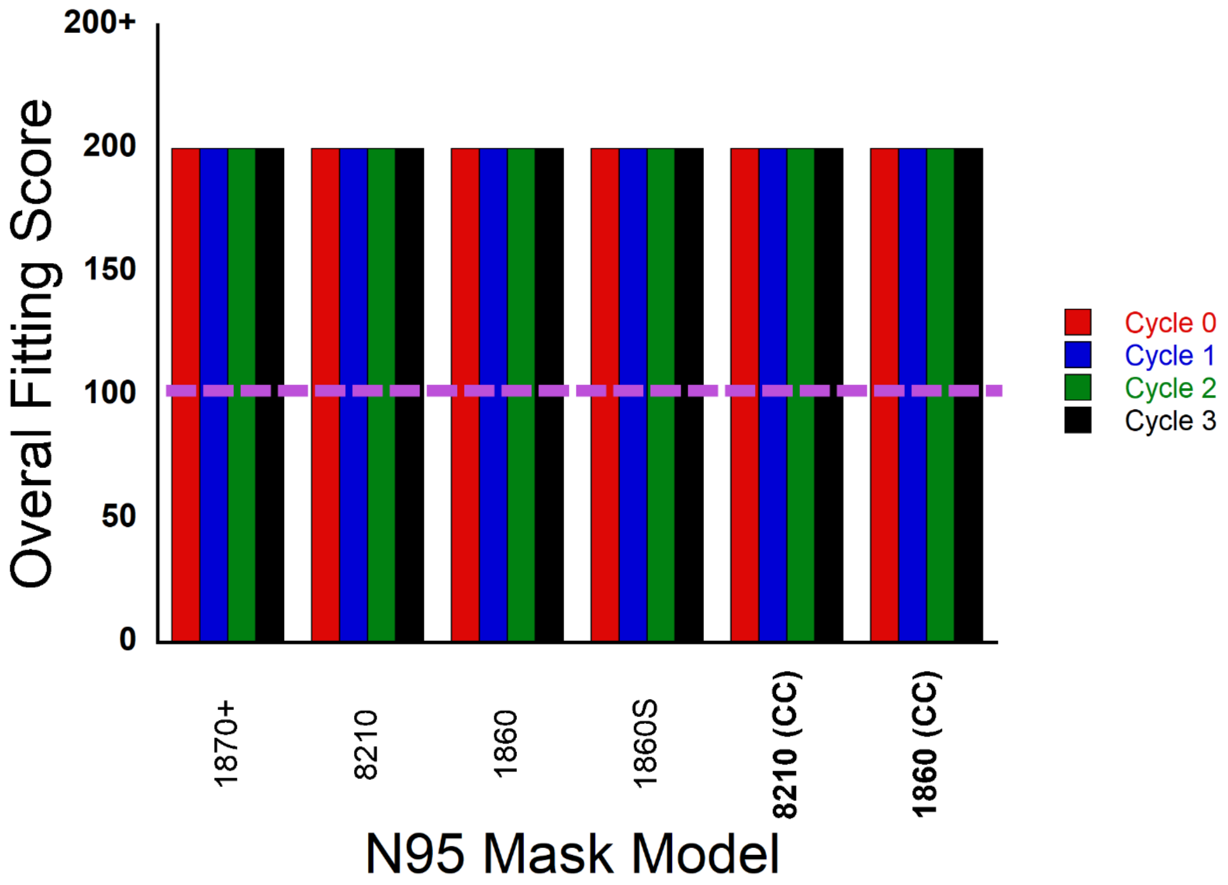

Figure S4. Fit testing of different N95 mask after variable number of decontamination cycles. The photothermal cycle was the white cycle, with the exception of the last 2 column groups that were used Color Cycle (CC). Overall Fitting Score after fit testing. A value of 100 or above is indicative of satisfactory test (limit highlighted with a purple straight arrow pointing to the right). Each type of mask was assessed for fit testing under the CSA standards (see methodology).

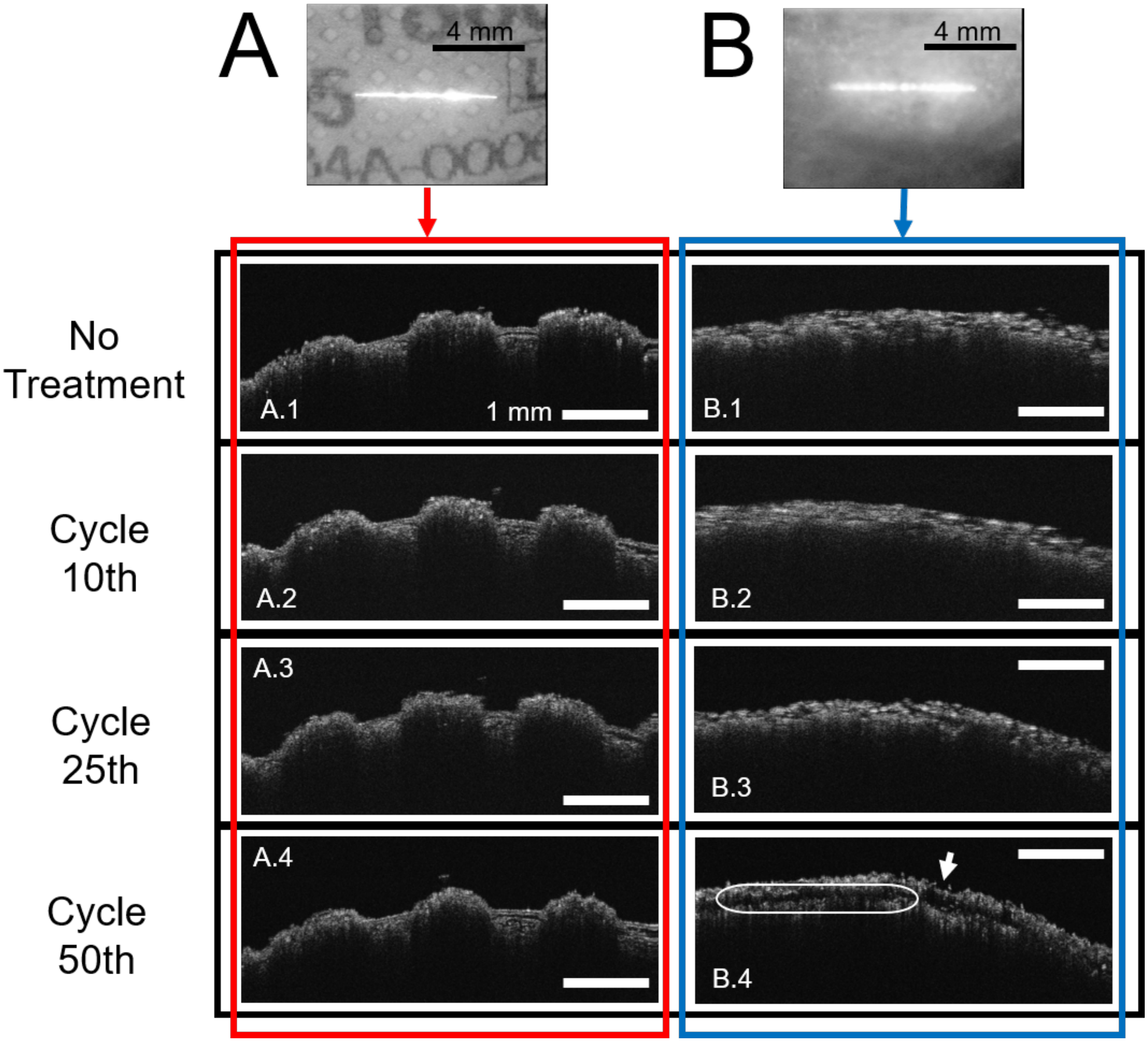

Figure S5. **Optical Coherence Tomography (OCT) of N95 masks after 50 treatment cycles. (A)** 1860S N95 (Color Cycle) and **(B)** 8110S N95 (White Cycle) masks evaluated through a horizontal linear OCT B-scan (5x2 line length/depth mm). At the top of every panel, the area of scan is highlighted. Each scan was measured at the same position in each condition. Arrows in 8110S N95 mask shows areas of significant deterioration. Ellipses indicate qualitative significant deterioration between the layers of the mask compared to the control.

Control strips (Absorbance at 600 nm is shown)			Position Inside the Chamber	White Cycle	Color Cycle
**Negative Control**	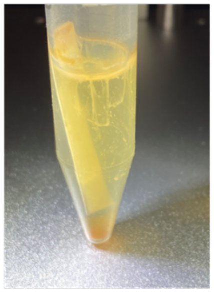<br>0.735		**Top**	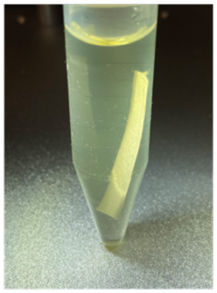<br>0.057	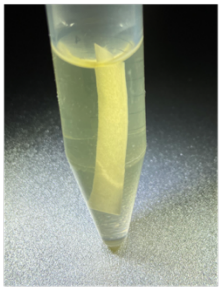<br>0.057
**Autoclave Control**	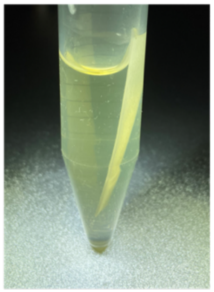<br>0.057		**Middle**	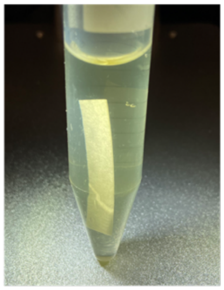<br>0.059	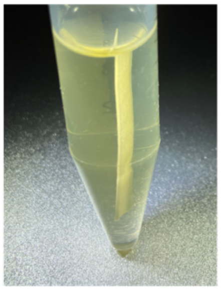<br>0.060
**Tryptic Soy Broth Control**	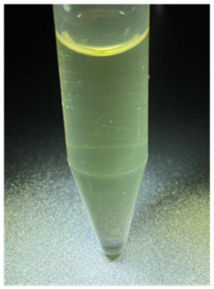<br>0.058		**Bottom**	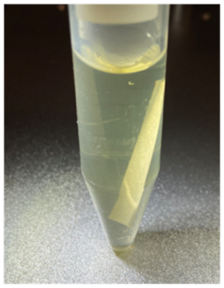<br>0.058	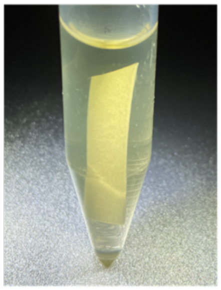<br>0.058

Figure S6. Spore strips qualitative quality control test. Spore strips of *Bacillus Pumilus* were used for the test (Biological Indicator Spore Strips, CROSSTEX, catalogue number: PM106, $2.2x10^6$ spores per strip). Each strip was placed in a custom-made hanger that was previously sterilized at different vertical positions of the rotating shaft in the device (same positions as the masks). After the evaluated cycle, the strips were transferred under sterile conditions to 5 mL of Trypsin Soy Broth, and incubated for 7 days at 37ºC, under aerobic conditions. After image acquisition, the tubes were homogenized, and 100 μL of each solution were transferred to a 96 well plate for absorbance measurement at 600 nm in a well plate reader (SpectraMax M2e, Molecular Devices). Negative Control = Strip with no treatment. Autoclave Control = Strip treated under 1 wet autoclave cycle. Tryptic Soy Broth = Sterile Broth with no further modification. White Cycle is 7 min UV + 12 min 60ºC, and Color Cycle is 10.5 min UV + 12 min 60ºC.

G. Stearothermophilus **test under only temperature treatment**		
**Groups**	**Representative image (after 12 h aerobic growth at 55ºC)**	**Absorbance at 600 nm, n=3**
**Tryptic Soy Broth**	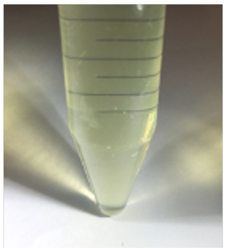	**0.059 ± 0.002**
**Negative Control (Strip with Bacteria)**	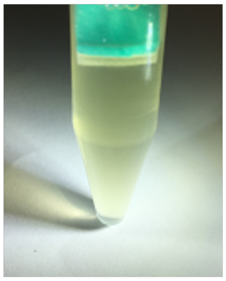	**0.445 ± 0.040**
**Strips without Bacteria after UV + Temperature treatment**	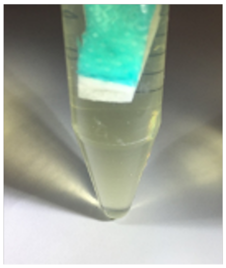	**0.057 ± 0.001**
**Strips with Bacteria after only Temperature treatment**	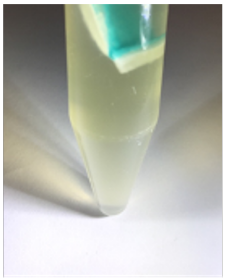	**0.479 ± 0.021**
**Strips with Bacteria after UV + Temperature treatment**	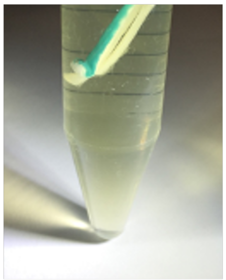	**0.060 ± 0.001**

Figure S7. Strips of the mask 1860S N95 mask were treated under different conditions with the bacteria *G. Stearothermophilus* in Tryptic Soy Broth. The test was performed under the protocol explained in the methodology. Post-treatment protocol modification: after the correspondent treatment condition and wait time, the strips were transferred to 5 mL of Tryptic soy Broth. This was cultured in aerobic conditions at 55 ºC and continues shaking. After 12 hours of incubation, an image acquisition of each tube was performed, the tubes were homogenized, and 100 μL of each solution were transferred to a 96 well plate for absorbance measurement at 600 nm in a well plate reader. Tryptic Soy Broth = Sterile Broth with no further modification. Negative Control = Strip with bacteria addition and no further treatment. For UV + temperature treatment, it was used a Color Cycle (10.5 min UV + 12 min 60ºC). For temperature treatment, it was used only 12 min at 60ºC in the device (no UV).

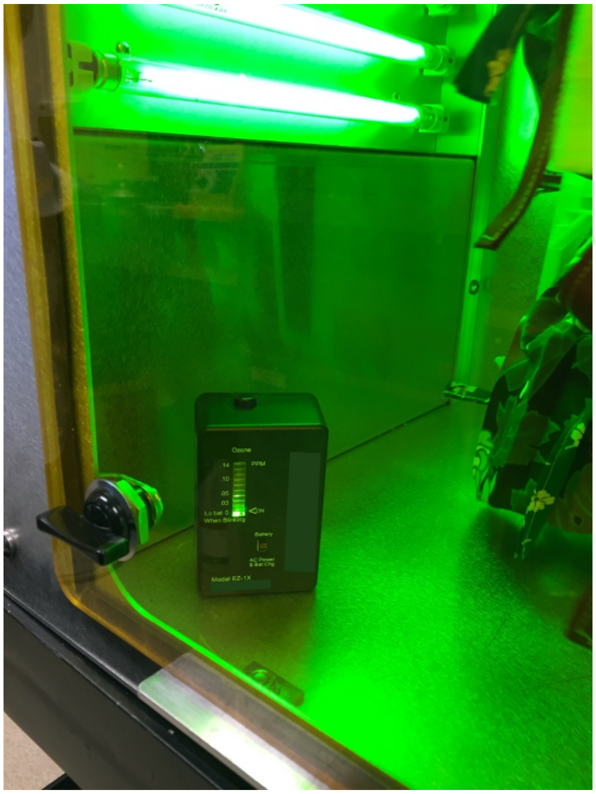

Figure S8. Representative image of the ozone measurements inside the device after 14 min of UV irradiation at the bottom level of the device.

CERTIFICATE OF CALIBRATION

MODEL NUMBER: EZ-1X

S/N: 816946

This document certifies that the instrument described above was calibrated in our facilities according to our standard procedure.

Ozone (ppm)	Tolerance (ppm)	PASS
< 0.03 (Low)	< 0.04	✓
0.1 (OSHA Standard Limit)	0.08 to 0.12	✓
>0.14 (High)	> 0.14	✓

Temperature (°F): **70.2**
Relative Humidity (%): **28**
Reference Analyzer: 732

Eco Sensors calibrated and verified this instrument using a reference standard UV ozone analyzer with a NIST traceable calibration. The calibration data for this instrument is stored in our permanent database.

The instrument is warranted to perform according to the specifications contained in the User Manual only if the instructions are carefully followed. Please contact us with any questions at sales@ecosensors.com or (800) 472-6626.

CALIBRATED BY: R. O
Ryan Ochoa

DATE OF CALIBRATION: 6/5/2020

Figure S9. Certificate of Calibration of the Ozone Monitor used in our study.

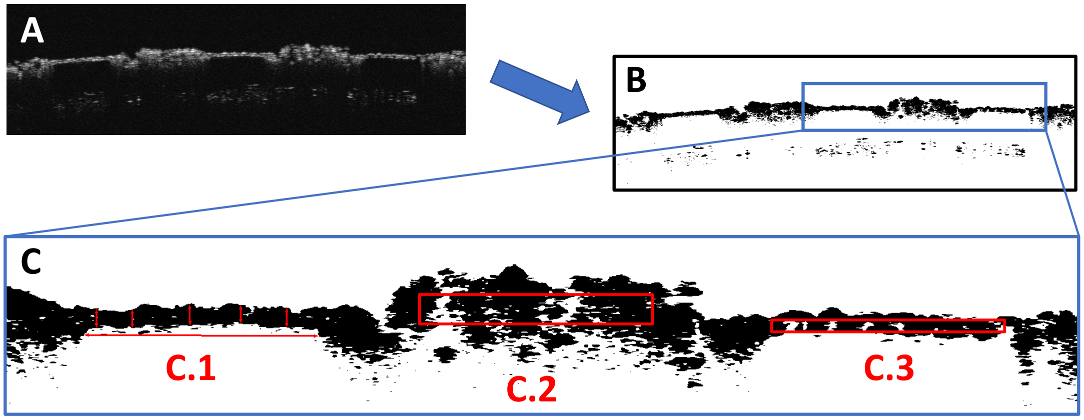

Figure S10. Image analysis process to obtain quantitative data from OCT. (A) original OCT image (7x2mm). **(B)** After thresholding the image. **(C)** Different sections evaluated in each OCT image; **C.1**: In a pore area of the mask, it was measured the thickness layer; **C.2**: a rectangle of 800x100 μm was drawn on a non-pore area and evaluate the density percentage of material; **C.3**: a rectangle of 800x40 μm was drawn on a layer at the pore area and evaluate the density percentage of material.

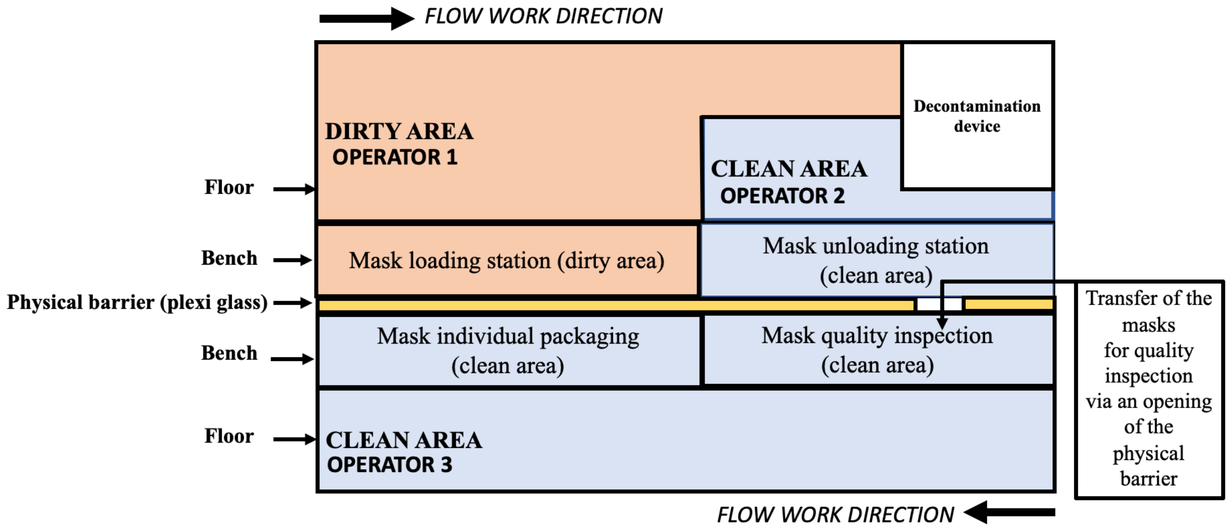

Scheme S1. Diagram depicting a proposed outline (used at the University of Ottawa Heart Institute facilities) for the decontamination process. To emphasize in this scheme is the fact that at least 3 operators must work in separated workstations. At all times operators must avoid crossing the dirty and clean areas. The arrows pointing as workflow direction indicate the direction where the masks must move during the decontamination process. This is a schematic representation and should be considered as guidance for the institutions operating the decontamination device.

Table S1. Physical inspection of N95 masks after 50 cycles of decontamination. Qualitative visual inspection was focused on signs of burns and deformation on mask 1870+ and 8110S (White Cycle), and 1860S (Color Cycle). The integrity of the elastic bands was carried out by stretching the bands from 2 to 6 cm. The odor inspection was done by the tester to identify ozone or burnt smells. The scoring rank was: 3 = No changes with respect to control (before decontamination), 2 = small changes, it does not compromise the physical integrity of the masks, 1 = significant changes (borderline), and 0 = not usable masks. (†) = 1-hour post-decontamination score came back to 3. Values of all the mask previous the first cycle are 3.

	**Cycle 10th**			**Cycle 25th**			**Cycle 50th**		
**Mask Model**	1870+	8110S	1860S	1870+	8110S	1860S	1870+	8110S	1860S
**Visual Inspection**	3	3	3	3	3	3	3	3	3
**Elastic band Test**	3	3	3	2.5	2.5	2.5	2.5	2.5	2.5
**Odor Inspection**	3	3	3	2.5 (†)	2.5 (†)	2.5 (†)	2	2	2

Table S2. Filtration efficiency percentage for decontaminated N95 masks (three cycles) measured under 85 L/min flow rate. * N95 1860 mask was carried out using the "coloured" decontamination cycle in the device.

Mask model	**Test Facility**	**Filtration**	**Flow**
		**%**	**L/min**
1870+	M36-B119	99.5	85
1870+	M36-B119	99.1	85
1870+	M36-B119	99.4	85
8210	M36-B119	99.4	85
8210	M36-B119	99.7	85
8210	M36-B119	99.8	85
*1860**	M36-406B	99.9	85
*1860**	M36-406B	100	85
*1860**	M36-406B	99.8	85

Table S3. Fit testing of different N95 masks after 50 decontamination cycles. Overall Fitting Score after fit testing was performed on the masks 1870+ and 8110S (White Cycle), and 1860S (Color Cycle). A value of 100 or above is indicative of satisfactory test. Each type of mask was assessed for fit testing under the CSA standards (see methodology).

	Cycle 0th			Cycle 10th			Cycle 25th			Cycle 50th		
**Mask Model**	1870+	8110S	1860S	1870+	8110S	1860S	1870+	8110S	1860S	1870+	8110S	1860S
**Overall Fit Factor**	200+	200+	199	200+	200+	192	188	200+	197	200+	200+	188

Table S4. Analysis of N95 first layer thickness and density by OCT after 50 disinfection cycles. Semi-quantitative measurements of the first layer of the 1860S N95 and 8110S N95 masks. 1860S mask integrity was assessed at the porous area (thickness and density) and at the non-porous area (density). 8110S mask integrity was assessed as the density at the first layer. For further details see methodology. * = significant difference ($p<0.05$), and ** = significant difference ($p<0.01$), compared to the respectively "No Treatment" results, calculated from t-test analysis (n=3).

**Mask model**		**1860S (Color Cycle)**			**8110S (White Cycle)**
		**Layer thickness at porous area (µm)**	**Density of first layer at porous area (%)**	**Density of first layer at non-porous area (%)**	**Density of first layer (%)**
Photothermal decontamination	No treatment	90.89 ± 13.53	97.12 ± 1.98	94.47 ± 1.92	91.45 ± 2.38
	10th Cycle	83.79 ± 16.07	91.17 ± 4.72	91.24 ± 3.73	86.13 ± 12.20
	25th Cycle	67.81 ± 12.04	87.07 ± 2.74	90.67 ± 0.37	92.97 ± 3.17
	50th Cycle	70.15 ± 8.89	96.09 ± 0.33	94.32 ± 0.95	82.17 ± 7.32

Table S5. Number of bacteria colonies counted before (control) and after the decontamination process. *Measured using colour cycle

Mask model (CFU/ML)				
**Bacteria Strain and Treatment Group**		1860	8210	1870+
***P. aeruginosa* (PA14)**	**Control**	$4.9 \pm 0.5 \times 10^{10}$	$4.9 \pm 0.5 \times 10^{10}$	$4.9 \pm 0.5 \times 10^{10}$
	**UV**	$2.3 \pm 0.48 \times 10^{5}$	0	$8.9 \pm 7.6 \times 10^{3}$
	**UV + 60ºC**	0	0	0
***S. epidermidis* (ATCC 35984)**	**Control**	$4.0 \pm 0.4 \times 10^{10}$	$4.0 \pm 0.4 \times 10^{10}$	$4.0 \pm 0.4 \times 10^{10}$
	**UV**	$1.4 \pm 0.2 \times 10^{5}$	0	0
	**UV + 60ºC**	$4.4 \pm 7.0 \times 10^{3}$	0	0
Geobacillus stearothermophilus	**Control**	$2.5 \pm 1.3 \times 10^{10}$	$2.5 \pm 1.3 \times 10^{10}$	$2.5 \pm 1.3 \times 10^{10}$
	**UV**	$1.8 \pm 0.8 \times 10^{6}$	$1.6 \pm 2.2 \times 10^{6}$	0
	**UV + 60ºC**	$4.4 \pm 7.0 \times 10^{3}$	$1.3 \pm 2.3 \times 10^{4}$	0
Geobacillus stearothermophilus*	**Control**	$3.8 \pm 0.8 \times 10^{10}$		
	**UV**	$1.1 \pm 1.8 \times 10^{5}$		
	**UV + 60ºC**	0		

Table S6. Viral titer (IU/mL) measured before (control) and after the decontamination process. *Measured using colour cycle.

Mask Model (Viral Titter, IU/mL)				
pLL-CMV-rFLuc-T2A-GFP-Puro LENTI-LABELER		**1860**	**8210**	**1870+**
	**Control**	$2.2 \pm 0.8 \times 10^9$	$1.1 \pm 0.4 \times 10^9$	$1.0 \pm 0.4 \times 10^9$
	**UV**	$2.1 \pm 0.5 \times 10^6$	$1.4 \pm 0.8 \times 10^5$	$8.0 \pm 2.2 \times 10^5$
	**UV + 60ºC**	$5.9 \pm 1.7 \times 10^5$	$4.4 \pm 3.0 \times 10^4$	$4.4 \pm 2.0 \times 10^4$
pLL-CMV-rFLuc-T2A-GFP-Puro LENTI-LABELER*	**Control**	$2.2 \pm 0.8 \times 10^9$		
	**UV**	$6.6 \pm 5.7 \times 10^4$		
	**UV + 60ºC**	$3.3 \pm 1.4 \times 10^4$		

Video S1. Videos of the device operation. Mask loading.

https://www.dropbox.com/s/5vfuhaxuqtr381w/Mask%20loading-Automated%20System.m4v?dl=0

Video S2. Videos of the device operation. Device operation.

https://www.dropbox.com/s/znwtuo1xwp8py3z/Device%20operation-Automated%20Version.m4v?dl=0
